# Notes and News

**Published:** 1904-05-21

**Authors:** 


### NOTES AND NEWS.

The Regius Professor of Physics, Cambridge, Dr. Clifford Allbutt, opened the new Public Dispensary at Leeds last week. It is claimed to be the finest of its kind in Great Britain, and cost £33,000.

Mr. A. E. Boycott, M.B., has made a report on the method of ascertaining the presence of ankylostomiasis, and this report has been published as a Parliamentary Paper. The report enters into full details as to the method of conducting the examination of, and the putting up of, specimens for microscopic work.

It is reported that the results of Dr. Koch's investigations of Bovine Plague in Egypt have disappointed the authorities. He has advised the Government to pursue the measures they have already adopted, whereas it was hoped that he would have advised new and more immediately effective measures.

Mr. Howard Marsh, M.A., F.R.C.S., Professor of Surgery in the University of Cambridge, will deliver the Hunterian Lecture at St. George's Hospital Medical School on May 25th, 1904, at 8.30. The lecture will include "Intermittent Hydrops of the Joints" and "The Influence of Growth on Deformities." Members of the medical profession are invited to attend.

Mr. Long has been approached by a deputation of the Royal Statistical Society and other bodies on the subject of a quinquennial census. This proposition is made in the interests of the health and wealth of the nation. Mr. Long's reply in brief amounted to a statement that if the Treasury saw fit to grant the large expenditure entailed that the proposal could be carried out. There can be no doubt that at the present time public attention has been so awakened upon the questions relating to the nation's health, wealth, and progress, that impatience is felt by many when figures are not available to witness to the correctness of many theories raised. But it is a question open to doubt whether a period of five years is sufficiently long to convincingly reveal the results of the habits and actions of the population for any serious and scientific purpose. If it cannot do this, it is too vast, unwieldy, and expensive an undertaking to be lightly entered into

The late Mrs. Salter, of Leith, has bequeathed £1,000 to the Aberdeen Royal Infirmary.

Lord Tredegar has offered to defray the cost of adding verandahs to the Cardiff Infirmary, and his offer has been accepted.

The funeral of Mr. Adrian Hope, secretary of the Hospital for Sick Children, Great Ormond Street, took place at Hopton, Suffolk, on Saturday. A memorial service was held in the chapel of the hospital.

The employees of 23 railway companies took part in the annual competition organised by the St. John's Ambulance Association this year; the competition included bandaging and stretcher work and a *viva voce* examination. The team representing the Great Eastern Railway won the shield, that of the Great Western Railway running them very close. This is the fourth occasion on which the Great Eastern have been successful. Each member of the winning team received a clock, and from Mr. Goodday, general manager of the railway, five pounds.

An interesting discussion on the respective merits of compulsory and voluntary support for our hospitals was held at the London School of Economics on May 12th. The majority of the students present voted for the compulsory maintenance of our hospitals by money drawn either from the rates or from imperial sources; but, strangely enough, Mrs. M'Killop, the lady who opened the discussion on this side, said finally that, while she wished hospitals to have State support, she wished also that voluntary hospitals should continue to exist. It is difficult to see in what the method she desires to see established would differ from the existing one. Miss Leigh, who ably defended the voluntary system, mentioned, as a proof of its efficiency, the success that had recently attended the efforts of the Salop Infirmary to add to the funds of the institution when they adopted the recommendations of Sir Henry Burdett.

The Metropolitan Asylums Board have received letters of protest from 12 Boards of Guardians against their proposal to permit experimental work in connection with the causation of small-pox. In connection with this subject, the Canine Defence League has addressed the Home Secretary in protest against granting the license to the Joyce Green Hospital. The wording of the address is very positive. It runs:—"The uselessness of such inoculative experiments has again and again been proved beyond all controversy. But, even if this were not so, my Society is of opinion that these experiments, by inflicting weeks of unrelieved torture upon sentient creatures, are in themselves so hideously cruel that no utilitarian considerations could justify them. We therefore, Sir, most earnestly beg that the licence to experiment may not be granted to this hospital, and that the authorities at the Lister Institute may be made to understand that their proposed experiments upon dogs would be a violation of the first principles of humanity, involving a violation of the right of the ratepayer to insist that the rates he has paid should be devoted exclusively to the purpose for which they have been levied."

